# Assessing biomarkers for predicting Alzheimer's disease in TBI patients: A subanalysis of the double‐blinded, phase III randomized clinical trial with biperiden

**DOI:** 10.1002/alz70856_103686

**Published:** 2025-12-25

**Authors:** Michele Longoni Calio, Luis E. Santos, Amanda Cristina Mosini, Maira Licia Foresti, Fernanda Guarino De Felice, Luiz Eugênio De Moraes Mello

**Affiliations:** ^1^ Universidade Federal do Rio de Janeiro, Rio de Janeiro, Rio de Janeiro, Brazil; ^2^ D'OR Institute for Research and Education IDOR, Rio de Janeiro, Rio de Janeiro, Brazil; ^3^ Universidade Federal de São Paulo, São Paulo, São Paulo, Brazil; ^4^ D'Or Institute for Research and Education, Rio de Janeiro, RJ, Brazil; ^5^ Queen's University, Kingston, ON, Canada; ^6^ Federal University of Rio de Janeiro, Rio de Janeiro, RJ, Brazil

## Abstract

**Background:**

Traumatic Brain Injury (TBI) is a significant risk factor for Alzheimer's Disease (AD). However, the mechanisms connecting TBI to AD pathology remain unclear. Identifying blood‐based biomarkers of neuronal and astrocytic injury is crucial for advancing precision diagnostics and intervention by predicting neurodegeneration. This study evaluates serum biomarkers using the ultra‐sensitive Single Molecule Array (SIMOA) platform to explore their association with TBI severity, sex‐specific responses and potential relevance to AD.

**Methods:**

We conducted a subanalysis of 123 patients enrolled in the phase III NCT01048138 clinical trial at HCFMUSP. Participants with acute TBI were randomized to receive either biperiden or placebo. Serum samples were collected and analyzed at multiple time points for biomarkers including Tau, NfL, GFAP, and UCHL1 using SIMOA. Biomarker profiles were compared across treatment groups, TBI severity, sex, age and time post‐injury. Additional analyses explored their relevance to AD‐related neurodegenerative process.

**Results:**

GFAP and NfL emerged as the most reliable biomarkers, strongly correlating with age, sex, TBI severity and temporal progression post‐injury. Intriguingly, elevated levels of these biomarkers in the acute phase post‐TBI were associated with astrocytic and axonal injury, which are critical in AD pathology. UCHL1 levels are also associated with trauma severity, sex, and time post‐injury, but less significantly than NfL and GFAP. Women exhibited higher levels of levels Tau, GFAP and UCHL1 but lower NfL levels compared to men, suggesting a potential sex‐specific response. Age‐stratified analyses revealed increased NfL and GFAP levels in older patients, emphasizing the impact of age on astrocytic activation. While biperiden treatment did not significantly alter biomarker levels across the overall cohort, exploratory analyses also revealed possible sex‐specific trends in treatment response.

**Conclusions:**

Our findings underscore the feasibility of serum biomarkers such as GFAP and NfL to bridge the understanding of the molecular link between TBI and AD. The SIMOA technology enables precise quantification of biomarkers, providing valuable knowledge into the time window from the moment of TBI to the development of AD, allowing for the analysis of long‐term neurodegeneration. These results reinforces the importance of personalized approaches to diagnosis and therapeutic monitoring.

